# PPP2CA knockdown upregulates the expression levels of ferroptosis-related genes TFRC and ACSL4 in colorectal cancer cells by promoting mTOR phosphorylation

**DOI:** 10.3389/fonc.2025.1694932

**Published:** 2025-11-06

**Authors:** Jing Cheng, Zhihan Liu, Haibo Liu, Chao Fang, Jun Li

**Affiliations:** 1 Department of Gastroenterology, Lianyungang Municipal Oriental Hospital, Lianyungang, Jiangsu, China; 2 Department of Gastroenterology, the Affiliated Lianyungang Oriental Hospital of Kangda College of Nanjing Medical University, Lianyungang, Jiangsu, China; 3 Department of General Surgery, the Affiliated Jiangning Hospital of Nanjing Medical University, Nanjing, Jiangsu, China; 4 Department of Central Laboratory, the Affiliated Jiangning Hospital of Nanjing Medical University, Nanjing, Jiangsu, China

**Keywords:** protein phosphatase 2A catalytic subunit alpha, colorectal cancer, ferroptosis, malignant phenotype, mTOR

## Abstract

Colorectal cancer (CRC) is one of the most prevalent malignant tumors worldwide, with its pathogenesis tightly linked to the regulation of cell death. Ferroptosis plays a pivotal role in the initiation, progression, and treatment of CRC. In our previous studies, we demonstrated that PPP2CA knockdown enhances the malignant phenotype of CRC cells while increasing their susceptibility to ferroptosis, albeit this latter effect frequently mitigates the malignant phenotype. The present study aims to further elucidate the regulatory mechanism underlying the association between PPP2CA and ferroptosis.​ Lentiviral-mediated PPP2CA knockdown was performed in CRC cell lines HCT116 and SW480 to establish stable PPP2CA-knockdown models, and PPP2CA gene expression in these models was verified. Colony formation assays and scratch wound healing assays were used to assess the proliferative and migratory capacities of CRC cells after PPP2CA knockdown. Additionally, CRC samples from The Cancer Genome Atlas (TCGA) database were analyzed via bioinformatics approaches to identify ferroptosis-related genes closely correlated with PPP2CA, with subsequent validation in model cells. The STRING database was employed to analyze the interaction networks between PPP2CA and target ferroptosis-related genes, predict potential signaling pathways via which PPP2CA regulates cellular ferroptosis, and validate these findings in model cells.​ Compared with the control group, HCT116 and SW480 cells in the PPP2CA-knockdown group exhibited significantly enhanced proliferative and migratory capacities. Bioinformatics analysis identified the top 10 most significant ferroptosis-related genes associated with PPP2CA. Validation in model cells showed that the expression levels of transferrin receptor (TFRC) and acyl-CoA synthetase long-chain family member 4 (ACSL4) were significantly upregulated. STRING analysis and in-model-cell validation indicated that increased mTOR phosphorylation subsequent to PPP2CA knockdown could upregulate the protein expression levels of transferrin receptor (TfR, encoded by the TFRC gene) and ACSL4, and this effect could be reversed by the mTOR inhibitor rapamycin.​ Thus, PPP2CA knockdown enhances the malignant phenotype of CRC cells, while potentially upregulating the expression of ferroptosis-related genes TFRC and ACSL4 via the mTOR signaling pathway, thereby increasing ferroptosis sensitivity.

## Highlights

- PPP2CA plays an important role in CRC ferroptosis.
- PPP2CA knockdown promotes CRC cell proliferation and migration.
- Identifies TFRC and ACSL4 as key ferroptosis genes.
- PPP2CA regulates CRC ferroptosis via mTOR pathway.

## 1 Introduction

Colorectal cancer (CRC) is the third most prevalent malignant neoplasm worldwide, with persistently high mortality that poses a substantial threat to human health (1). Therefore, investigating the pathogenesis of CRC and developing novel, effective therapeutic strategies is particularly important. Protein phosphorylation modification encompasses both phosphorylation and dephosphorylation, exerting a critical regulatory role in tumorigenesis and progression (2). Protein phosphorylation involves the transfer of phosphate groups to amino acid residues of target proteins, catalyzed by protein kinases, whereas dephosphorylation is the reverse process, catalyzed by protein phosphatases.​ Protein phosphatase 2A (PP2A) is a serine/threonine phosphatase that exists as a trimer consisting of structural, catalytic, and regulatory subunits. It participates in multiple critical biological processes, including apoptosis, autophagy, proliferation, and DNA repair (3). PP2Acα, encoded by the PPP2CA gene, serves as the catalytic subunit of the PP2A complex. Studies have demonstrated that it regulates the development and progression of multiple cancers, including hepatocellular carcinoma and gastric cancer (4, 5). In CRC, studies suggest that low PPP2CA expression may correlate with poorer overall survival in patients (6). Our previous studies have also confirmed significant downregulation of PPP2CA expression in CRC (7).

Ferroptosis, a form of regulated cell death, plays a critical role in the initiation and progression of various tumors, including CRC (8). Studies have shown that knockdown of methyltransferase-like protein 17 (METTL17) enhances the sensitivity of CRC cells to ferroptosis inducers, thereby inhibiting cell proliferation, migration, and invasion (9). Additionally, knockout or inhibition of solute carrier family 7 member 11 (SLC7A11) can induce ferroptosis, leading to the selective death of CRC cancer stem cells (10). Notably, in CRC cells with PPP2CA knockdown, a more prominent malignant phenotype was observed, accompanied by increased sensitivity to ferroptosis. This phenomenon may be attributed to the promotion of AMP-activated protein kinase (AMPK) dephosphorylation, which subsequently reduces the expression level of stearoyl-CoA desaturase 1 (SCD1). Consequently, this enhances the sensitivity of cells to ferroptosis while simultaneously augmenting the malignant phenotype of CRC cells (7). PPP2CA exerts tumor-suppressive effects, and its knockdown increases the sensitivity of cells to ferroptosis. However, heightened sensitivity to ferroptosis typically predicts a reduction in the malignant phenotype of cells. This apparent contradiction requires further investigation. The biological behavior of tumor cells is regulated through highly complex mechanisms, and the mechanisms underlying ferroptosis are equally intricate (11). To further explore the potential association between PPP2CA and ferroptosis, we intend to screen for ferroptosis-related genes potentially linked to PPP2CA using bioinformatics approaches and analyze the molecular mechanisms using cell models.

## 2 Materials and methods

### 2.1 Materials

All cell experiments were performed in adherence to the Declaration of Helsinki. All experimental procedures were approved by the Animal Welfare Committee of Nanjing Medical University and performed in line with standard protocols.

Fetal bovine serum (cat. no. A5669701), DMEM high-glucose medium (cat. no. 11965092), and trypsin (cat. no. 25200072) were purchased from Gibco (USA). Puromycin (cat. no. ST551), modified Giemsa stain solution (20×, cat. no. C0131), and RNA extraction kit (cat. no. R0017S) were obtained from Shanghai Biyuntian Co., Ltd. cDNA reverse transcription kit (cat. no. R323-01) and amplification kit (cat. no. Q341-02) were sourced from Nanjing Vazyme Biotech Co., Ltd.

The anti-PP2AC antibody (cat. no. 13482-1-AP) and anti-GAPDH antibody (cat. no. 10494-1-AP) were acquired from Wuhan Sanying Biotechnology Co., Ltd. Additionally, the anti-mTOR (phospho Ser2448) antibody (cat. no. YP-Ab-14329), anti-ACSL4 antibody (cat. no. YP-Ab-12589), and anti-TfR antibody (cat. no. YP-Ab-14016) were purchased from Hangzhou Zhenyoupin Biotechnology Co., Ltd. Rapamycin (cat. no. R485503) was obtained from Shanghai Aladdin Biochemical Technology Co., Ltd.

The cell lines used in this study were HCT116 and SW480, which were obtained from the American Type Culture Collection (ATCC).

### 2.2 Bioinformatics analysis

For this analysis, the data used were derived from TCGA. This study utilized the UALCAN (University of Alabama at Birmingham Cancer Data Analysis Portal) online analysis platform to analyze the expression levels of these 60 ferroptosis-related genes in tumor tissues and normal tissues, focusing on two major subtypes of colorectal cancer: READ (Rectal Adenocarcinoma) and COAD (Colorectal Adenocarcinoma).​ Specifically, the READ dataset included 10 cases of normal rectal tissues and 166 cases of primary READ tumor tissues, while the COAD dataset contained 41 cases of normal colorectal tissues and 286 cases of primary COAD tumor tissues. The Search Tool for the Retrieval of Interacting Genes/Proteins (STRING; http://cn.string-db.org) was employed to analyze the interaction relationships among key proteins involved in regulatory processes.

### 2.3 Cells culture

All cells were cultured in a constant-temperature incubator at 37 °C with 5% CO_2_, and maintained in complete Dulbecco’s Modified Eagle Medium (DMEM) supplemented with 1% double antibiotics and 10% fetal bovine serum (FBS). For cell passaging, 0.5% trypsin was used for digestion. Cells in the knockdown group were treated with the mammalian target of rapamycin (mTOR) pathway inhibitor rapamycin (RAPA) at a concentration of 100 nmol/L for 24 hours, which served as the knockdown treatment group (shPPP2CA+RAPA).

### 2.4 Cells infection

HCT116 and SW480 cells were divided into control and gene knockdown groups. The control group was infected with negative control lentivirus (shCon), while the knockdown group was infected with lentivirus targeting the PPP2CA gene (shPPP2CA), both at a multiplicity of infection (MOI) of 10.​ After 48 hours, the cells underwent puromycin selection (final concentration: 2 μg/mL), after which verification was performed to establish PPP2CA-knockdown HCT116 and SW480 cell lines for subsequent experiments.​ PPP2CA knockdown lentivirus was synthesized by Shanghai JiMan Biotechnology Co., Ltd. The shRNA sequence used in the experiment is as follows: 5′-GATCCGGCAAATCACCAGATACAAATTTCAAGAGAATTTGTATCTGGTGATTTGCCTTTTTTG-3′.

### 2.5 qRT-PCR detection

Cells in the logarithmic growth phase with 80% confluence were selected, and total RNA was isolated using an RNA extraction kit. The purity and concentration of RNA were assessed, and appropriate RNA samples were selected for reverse transcription to generate complementary DNA (cDNA) based on the concentration. The PCR amplification conditions were as follows: initial denaturation at 95°C for 30 seconds, followed by 40 cycles of denaturation at 95°C for 10 seconds, annealing at 60°C for 30 seconds, and extension at 72°C for 44 seconds, with a final extension at 63°C for 7 minutes. A 20 µL amplification system was prepared according to the kit instructions. The primer sequences are provided below, and the primers were synthesized by Nanjing GenScript (**Table 1**). Relative quantitative analysis was performed using the 2^-ΔΔCt^ method.

**Table 1:** Primer sequence.

Primer sequence
GADPH-F: 5′-GGAGCGAGATCCCTCCAAAAT-3′
GADPH-R: 5′-GGCTGTTGTCATACTTCTCATGG-3′
PPP2CA-F: 5′-GGTGGTCTCTCGCCATCTATAG-3′
PPP2CA-R: 5′-CTGGATCTGACCACAGCAAGTC-3′
TFRC-F: 5′-GGCAAGTAGATGGCGATAAC-3′
TFRC-R: 5′-ACAATAGCCCAAGTAGCCAA-3′
ACSL4-F: 5′-TTATTTTGCTGCCTGTCCAC-3′
ACSL4-R: 5′-AGCTAGTGAGTCGAAGTGTG-3′
FANCD2-F: 5′-AAAATTCCCAGGAGAGCACA-3′
FANCD2-R: 5′-TCTCCAGCACTTACTTCGTC-3′
GCLC-F: 5′-CATCCTACCCTTTGGAGACC-3′
GCLC-R: 5′-CTTGTTAAGGTACTGGGAAATGAA-3′
SLC7A11-F: 5′-ATTGGCTTCGTCATCACTCT-3′
SLC7A11-R: 5′-TTCTTCTGGTACAACTTCCAGT-3′
GCLM-F: 5′-GCGAGGAGGAGTTTCCAGA-3′
GCLM-R: 5′-TTCTACAATGAACAGTTTTGCAG-3′
IREB2-F: 5′-GTAGGAGTGGCTGGAAAGTT-3′
IREB2-R: 5′-GATTCGAGTTTGGCTTTGCT-3′
ACSL3-F: 5′-CACTGTTGATGGAAAGCCAC-3′
ACSL3-R: 5′-TGGTTTGCTATGAGGTTGGT-3′
NFE2L2-F: 5′-AGCACATCCAGTCAGAAACCA-3′
NFE2L2-R: 5′-CCTCATTGTCATCTACAAACGG-3′
NCOA4-F: 5′-TGCAGTTTGTGATCTCTTTGC-3′
NCOA4-R: 5′-AATTCCCAACGGTTACATCTTG-3′

### 2.6 Clonogenic assay

Cells were seeded into 6-well plates at a density of approximately 2000 cells per well and incubated in a constant-temperature cell culture incubator at 37 °C with 5% CO_2_ for 14 days. Subsequently, 2 mL of 4% paraformaldehyde was added to each well to fix the cells for 30 minutes. After fixation, cells were stained with 1× modified Giemsa working solution (diluted with double-distilled water) and incubated for 30 minutes. Following staining, the wells were washed three times with phosphate-buffered saline (PBS), dried, and observed under a microscope for photography. Images were analyzed using ImageJ software, and quantitative counting of formed colonies was performed. The formula for calculating the colony formation rate is as follows: Colony formation rate = Number of formed colonies/Number of seeded cells.

### 2.7 Scratch assay

Cells were seeded in 6-well plates at a density of 1 × 10^6^ cells/well and incubated at 37 °C in a 5% CO_2_ incubator. Once cells reached confluence (monolayer formation), a linear scratch was created by vertically scraping the monolayer with a sterile 200 μL pipette tip. Cells were gently rinsed three times with phosphate-buffered saline (PBS).​ The scratch healing process was observed and photographed under a microscope at 0, 24, and 48 h. Scratch width was measured and area was calculated using ImageJ software. Cell scratch migration rate was calculated via the formula: Cell scratch migration rate = (Scratch area at 0 h - Scratch area at 24 h or 48 h)/Scratch area at 0 h.

### 2.8 Western blot assay

Cell samples at approximately 80% confluency in the logarithmic growth phase were selected and lysed with RIPA lysis buffer. Total protein concentration was determined via the BCA assay. A 10% SDS-PAGE gel was prepared, and 10 μg of protein was loaded for electrophoresis.​ Proteins were transferred onto a PVDF membrane, which was then blocked for 2 h. Primary antibody was diluted per the manufacturer’s instructions and incubated with the membrane at 4 °C overnight. Following membrane washes, secondary antibody incubation was performed for 2 h, followed by further washes.​ The membrane was developed with ECL, and gray values were quantified using ImageJ software.

### 2.9 Statistical analysis

All experiments in this study were independently repeated three times. Experimental data are presented as mean ± standard deviation (Mean ± SD). All statistical analyses were performed using GraphPad Prism 9.0 software. Correlation analysis was conducted using the Pearson method, and the False Discovery Rate (FDR) was applied for multiple testing correction during gene screening. Independent samples *t*-test was used for between two groups comparisons, and One-way ANOVA was used for comparison among the three groups of samples, with *P* < 0.05 considered statistically significant.

## 3 Results

### 3.1 Knockdown of PPP2CA promotes proliferation and migration of CRC cell lines HCT116 and SW480​

**Figure 1:**
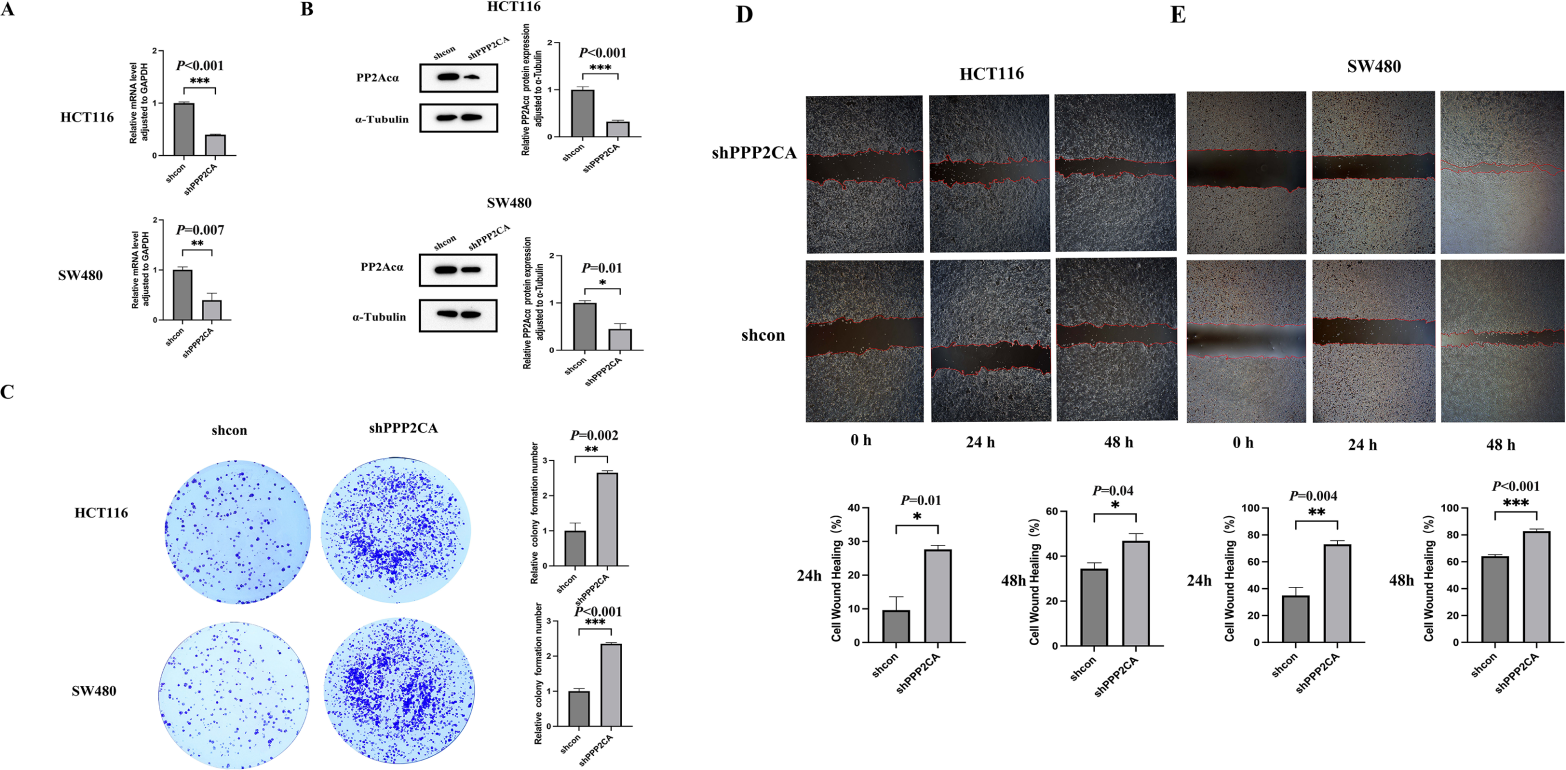
The knockdown efficiency of PPP2CA in HCT116 and SW480 and its impact on proliferation and migration capabilities **(A, B)** Construction of knockdown efficiency in HCT116 and SW480 knockdown groups; **(C)** Proliferation ability of HCT116 and SW480 knockdown groups and control groups; **(D, E)** Migration ability of HCT116 and SW480 knockdown groups and control groups *P < 0.05, **P < 0.01, ***P < 0.001. n=3, Mean ± SD.

PPP2CA mRNA and protein expression levels in HCT116 and SW480 cells with PPP2CA knockdown (shPPP2CA) were determined via quantitative reverse transcription polymerase chain reaction (qRT-PCR) and Western blot analysis. Results demonstrated that, compared with the control group (shcon), both PPP2CA mRNA and protein expression levels were significantly reduced in the shPPP2CA group (**Figures 1A, B**).​ Additionally, colony formation assays showed that the shPPP2CA (knockdown) groups of HCT116 and SW480 cells exhibited higher proliferative capacity than the control group (shcon) (**Figure 1C**). Furthermore, scratch assay results showed that the shPPP2CA (knockdown) groups of HCT116 and SW480 cells exhibited significantly increased cell migration rates at 24 and 48 h compared with the control group (shcon) (**Figures 1D, E**).

### 3.2 Screening of ferroptosis-related genes and analysis of their expression levels in CRC tissues​

We identified a total of 60 ferroptosis-related genes through a literature review (**Table 2**) (12–14). Based on the TCGA (The Cancer Genome Atlas) public cancer genomics database, this study utilized the UALCAN (University of Alabama at Birmingham Cancer Data Analysis Portal) online analysis platform to analyze the expression levels of these 60 ferroptosis-related genes in tumor tissues and normal tissues, focusing on two major subtypes of colorectal cancer: READ and COAD. Specifically, the READ dataset included 10 cases of normal rectal tissues and 166 cases of primary READ tumor tissues, while the COAD dataset contained 41 cases of normal colorectal tissues and 286 cases of primary COAD tumor tissues. The expression levels of each gene were compared between normal tissues and primary tumor tissues, with *P* < 0.05 considered statistically significant. Our results showed that 48 of these genes exhibited consistent expression patterns in both types of tumor tissues relative to normal tissues, with statistically significant differences in expression levels detected in at least one type of tumor tissue (**Table 2**). Subsequently, the correlation between PPP2CA and the expression levels of these 48 genes was analyzed using CRC samples from the TCGA database (**Table 3**). Using a significance threshold of *P* < 0.05, the correlation coefficients (r values) were sorted in descending order, and the top 10 ferroptosis-related genes were identified, which are as follows: FANCD2, GCLC, TFRC, ACSL4, SLC7A11, GCLM, IREB2, ACSL3, NFE2L2, and NCOA4.

**Table 2:** Analysis of the expression levels of 60 ferroptosis-related genes in tumor tissues (colon cancer and rectal cancer) and normal tissues from the TCGA database.

Ferrotosis-related genes	COAD		READ	
	*P* value	Expression in tumor	*P* value	Expression in tumor
ABCC1	1.62×10^−12^	Up	0.0000159	Up
ACACA	1.62×10^−12^	Up	0.0102	Up
ACO1	0.0032	Down	0.149	Down
ACSF2	1.75×10^−12^	Down	0.166	Down
ACSL3	4.45×10^−9^	Up	8.27	Down
ACSL4	1××10^−12^	Up	0.104	Up
AIFM2	0.418	Down	0.237	Up
AKR1C1	0.000000244	Down	0.0043	Down
AKR1C2	4.32×10^−12^	Down	0.202	Down
AKR1C3	1.31×10^−12^	Down	0.907	Down
ALOX12	0.0000618	Down	0.295	Down
ALOX15	0.0321	Down	0.0551	Up
ALOX5	0.00294	Down	0.0399	Down
ATP5MC3	1.11×10^−16^	Down	0.0000197	Down
CARS	1×10^−12^	Up	0.000057	Up
CBS	0.825	Down	0.000837	Up
CD44	1.62×10^−12^	Up	0.000000147	Up
CHAC1	1×10^−12^	Up	1.77×10^−12^	Up
CISD1	0.594	Up	0.0309	Down
CRYAB	0.000155	Down	0.00324	Down
CS	0.00588	Down	0.536	Down
DPP4	0.448	Down	0.175	Down
EMC2	1.62×10^−12^	Up	0.565	Down
FADS2	1.63×10^−12^	Up	0.000000242	Up
FANCD2	1.62×10^−12^	Up	0.000000599	Up
FDFT1	0.000000201	Up	0.905	Up
FTH1	3.33×10^−15^	Down	0.147	Down
G6PD	1×10^−12^	Up	0.000000184	Up
GCLC	1.1×10^−13^	Up	0.458	Up
GCLM	0.0000174	Up	0.000000742	Up
GLS2	1×10^−12^	Up	1×10^−12^	Up
GOT1	1.55×10^−15^	Down	0.000000855	Down
GPX4	0.0000167	Up	2.75×10^−13^	Up
GSS	1.63×10^−12^	Up	1.41×10^−9^	Up
HMGCR	0.0765	Down	0.51	Up
HMOX1	8.7×10^−12^	Down	0.0144	Down
HSBP1	0.0000219	Up	0.698	Up
HSPB1	0.266	Down	0.688	Down
IREB2	0.0199	Down	0.0425	Down
KEAP1	3.13×10^−12^	Up	0.00000406	Up
LPCAT3	6.84×10^−12^	Down	0.0604	Down
MT1G	1.72×10^−11^	Down	0.000883	Down
NCOA4	1×10^−12^	Down	1.62×10^−12^	Down
NFE2L2	0.0382	Down	0.12	Down
NFS1	1×10^−12^	Up	7.18×10^−12^	Up
NOX1	8.05×10^−13^	Up	2.06E-08	Up
NQO1	1.37×10^−14^	Up	1.91×10^−12^	Up
PEBP1	0.406	Up	0.899	Down
PGD	1.62×10^−12^	Up	0.00132	Up
PHKG2	0.0000188	Up	1.31×10^−8^	Up
PTGS2	0.000196	Up	0.179	Down
RPL8	1×10^−12^	Up	0.000000988	Up
SAT1	0.000196	Up	0.0114	Up
SLC1A5	1×10^−12^	Up	5.54×10^−8^	Up
SLC7A11	1.11×10^−16^	Up	4.36×10^−9^	Up
SQLE	1×10^−12^	Up	0.00826	Up
STEAP3	0.377	Down	0.00522	Up
TFRC	1.32×10^−8^	Up	0.128	Up
TP53	1.62×10^−12^	Up	2.45×10^−12^	Up
ZEB1	0.0032	Down	0.0141	Down

**Table 3:** Analyze the correlation between PPP2CA and the expression levels of 60 ferroptosis-related genes in CRC samples from the TCGA database.

Ferrotosis-related genes	Pearson *r*	Ferrotosis-related genes	Pearson *r*	Ferrotosis-related genes	Pearson *r*
GPX4	-0.20989	CHAC1	0.038654	HSBP1	0.366978
ALOX12	-0.20239	ABCC1	0.038913	CS	0.372116
ACSF2	-0.18713	TP53	0.071142	SAT1	0.387073
FTH1	-0.17919	GLS2	0.072909	ATP5MC3	0.405697
G6PD	-0.17538	AKR1C3	0.106887	CD44	0.420165
RPL8	-0.17058	ACACA	0.121794	GOT1	0.432772
SLC1A5	-0.16836	PGD	0.122237	FANCD2	0.461056
KEAP1	-0.16734	HMOX1	0.152234	GCLC	0.467069
PHKG2	-0.1479	NOX1	0.158213	TFRC	0.467627
GSS	-0.138	LPCAT3	0.207592	ACSL4	0.481241
MT1G	-0.05172	ZEB1	0.215823	SLC7A11	0.49096
NFS1	-0.02101	ACO1	0.228775	GCLM	0.578302
CRYAB	0.013489	NQO1	0.306738	IREB2	0.586031
FADS2	0.018182	SQLE	0.333801	ACSL3	0.589761
AKR1C1	0.023724	FDFT1	0.336136	NFE2L2	0.611206
ALOX5	0.027901	CARS	0.338518	NCOA4	0.696983

### 3.3 PPP2CA knockdown promotes upregulation of mRNA expression of ferroptosis-related genes TFRC and ACSL4 in CRC cells​

**Figure 2:**
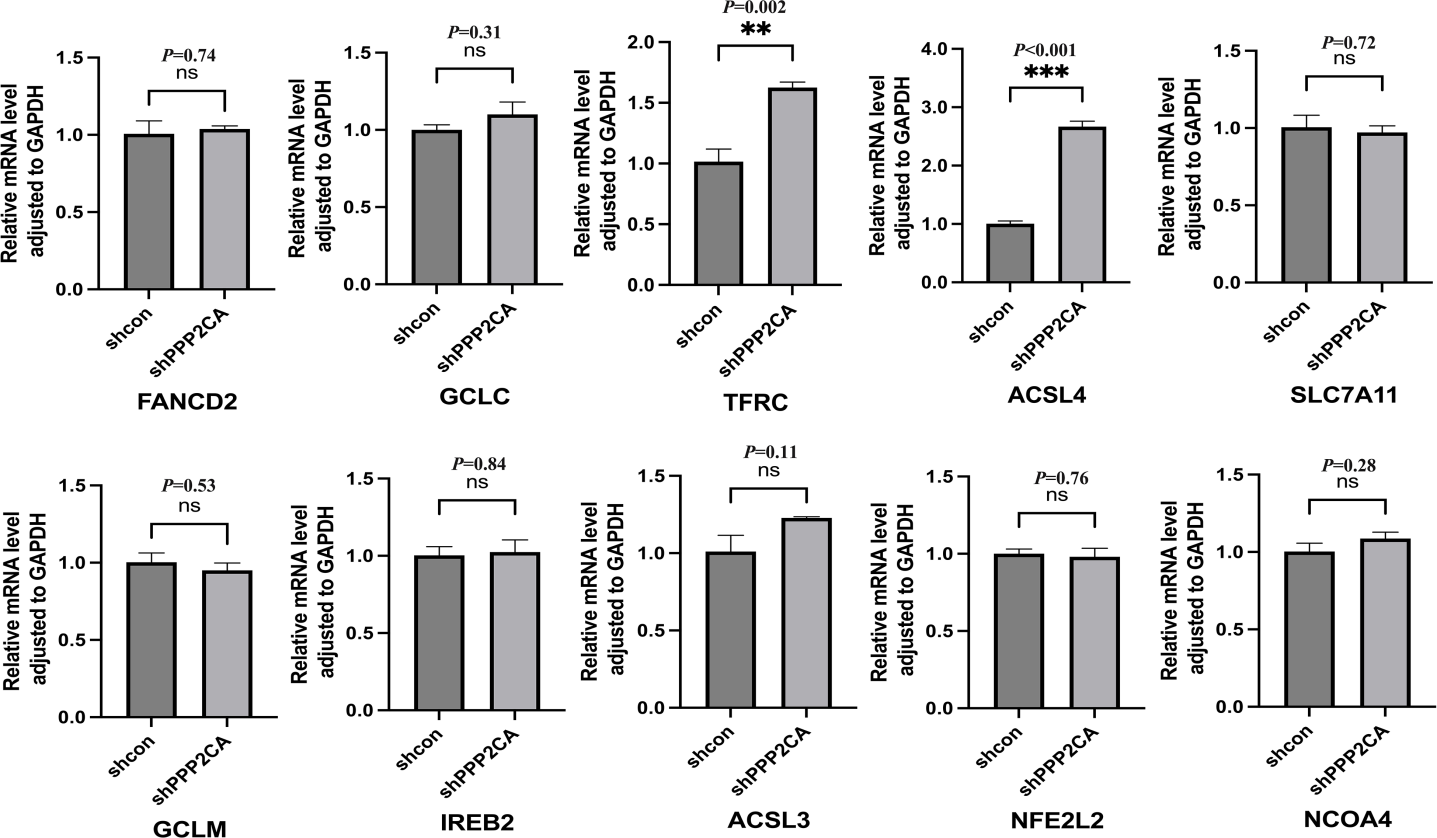
The mRNA expression levels of ferroptosis-related genes after PPP2CA knockdown. **P < 0.01, ***P < 0.001, ns: not significant. n=3, Mean ± SD.

mRNA expression of ten ferroptosis-related genes was detected in HCT116 cells of the shPPP2CA (knockdown) and shcon (control) groups via quantitative reverse transcription polymerase chain reaction (qRT-PCR). Results demonstrated that expression of transferrin receptor (TFRC) and acyl-CoA synthetase long-chain family member 4 (ACSL4) was significantly upregulated in the shPPP2CA group. However, no significant differences were noted in the expression of other ferroptosis-related genes between the two groups (**Figure 2**).

### 3.4 STRING database analysis of the interaction between PPP2CA and proteins encoded by TFRC and ACSL4​

**Figure 3:**
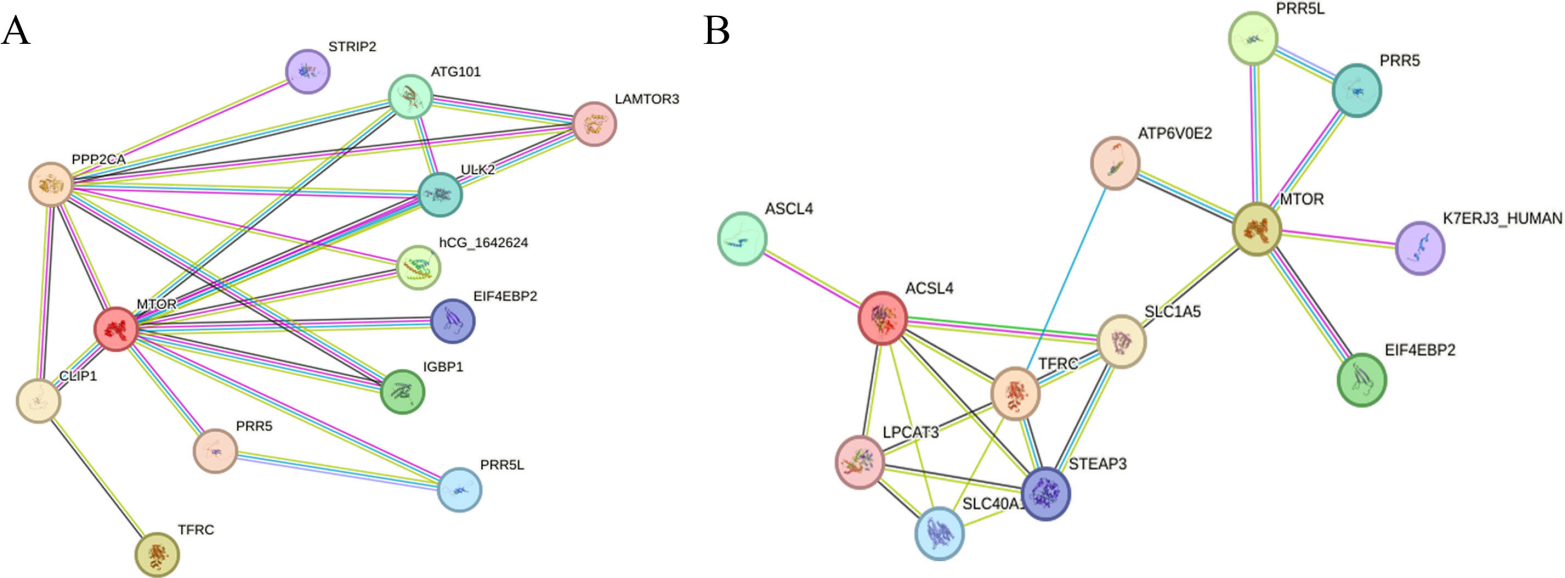
Analyzing the interaction relationships between proteins encoded by related genes in the STRING database **(A)** The interaction relationships among PPP2CA, TFRC, and mTOR; **(B)** The interaction relationships among mTOR, ACSL4, and TFRC.

To further analyze the potential regulatory relationship between PPP2CA and the proteins encoded by TFRC and ACSL4, we performed an analysis using the STRING database. Results showed that PPP2CA may be associated with TFRC via the mammalian target of rapamycin (mTOR) (**Figure 3A**), while mTOR interacts with the proteins encoded by both ACSL4 and TFRC (**Figure 3B**).

### 3.5 PPP2CA knockdown increases p-mTOR, TfR, and ACSL4 protein expression​

**Figure 4:**
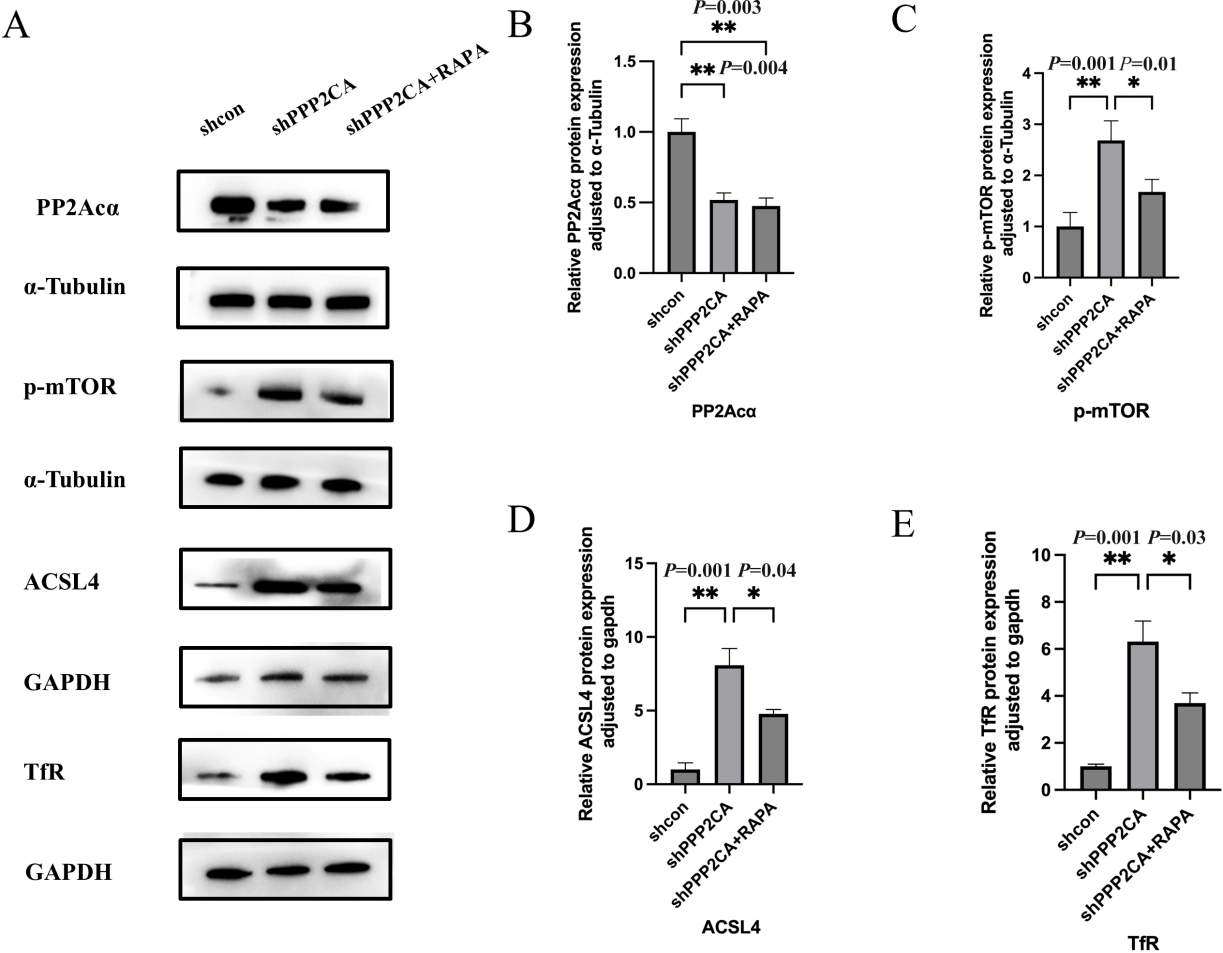
The expression levels of PP2Acα, p-mTOR, Acsl4, and TfR in HCT116 CRC cell lines **(A)** Protein electrophoresis image; **(B-E)** Protein quantification images of PP2Acα, p-mTOR, Acsl4, and TfR, respectively *P < 0.05, **P < 0.01. n=3, Mean ± SD.

**Figure 5:**
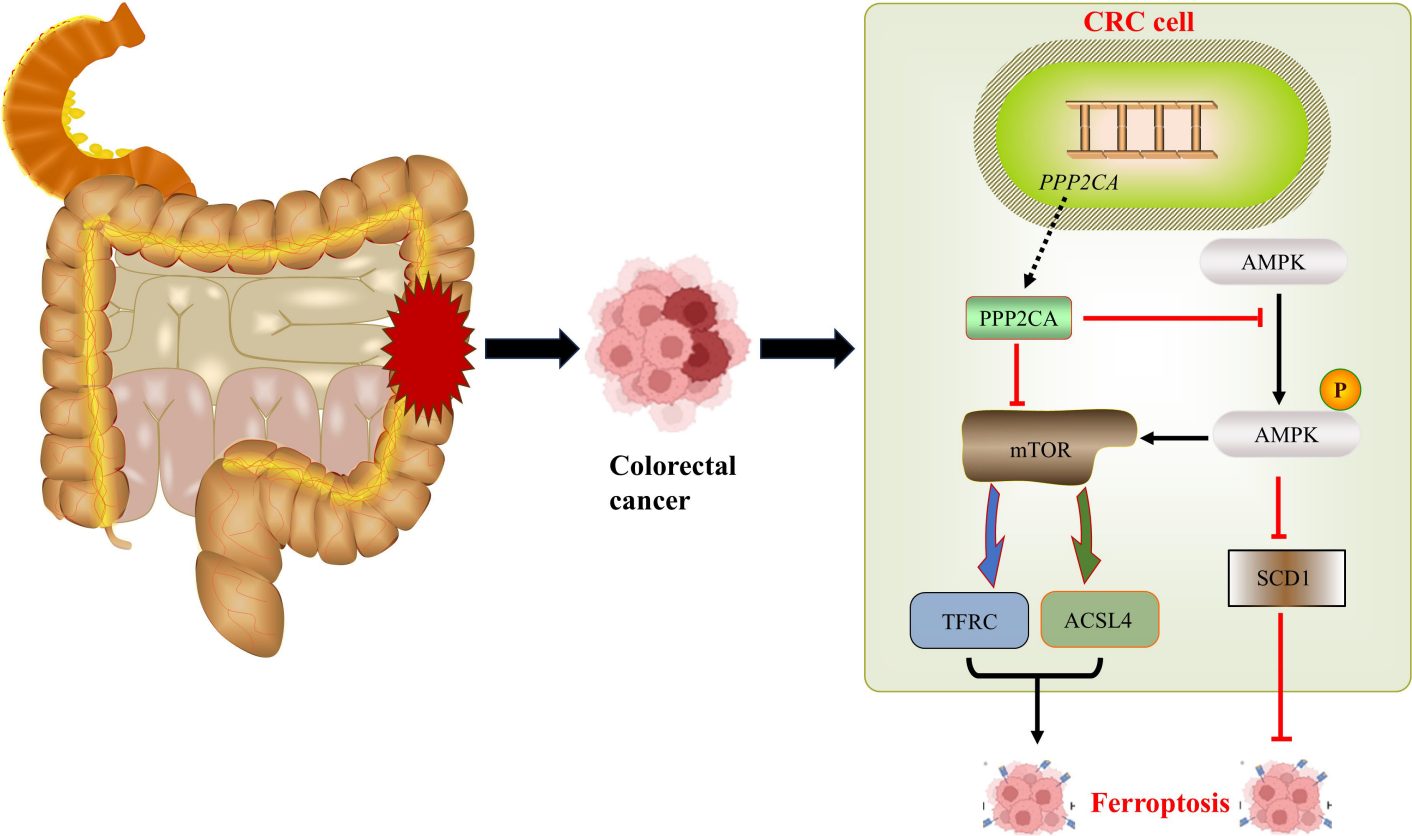
Signaling pathway diagram.

In the CRC cell line HCT116, PPP2CA knockdown increased TFRC and ACSL4 expression. To further validate these findings, cells in the knockdown group were treated with the mTOR pathway inhibitor RAPA (100 nmol/L), and Western blot analysis was subsequently performed to evaluate the expression of p-mTOR, TfR, and ACSL4 across groups.​ Results showed that p-mTOR, TfR, and ACSL4 expression was elevated in the shPPP2CA (knockdown) group compared with the shcon (control) group. Conversely, p-mTOR, TfR, and ACSL4 expression was decreased in the shPPP2CA+RAPA (knockdown plus RAPA treatment) group compared with the shPPP2CA group (**Figures 4A-E**, **5**).

## 4 Discussion

In recent years, despite significant advancements in colorectal cancer (CRC) treatment, both recurrence and mortality rates in patients remain high (15). Therefore, comprehensive exploration of the molecular mechanisms underlying CRC development and progression, along with further development of corresponding therapeutic strategies, is crucial for improving patient prognosis. In our previous studies, we found that the malignant phenotype of the CRC cell line HCT116 was significantly enhanced after PPP2CA knockdown (7). In the present study, we included the CRC cell line SW480 as a research subject. By using lentivirus to knock down the PPP2CA gene in these cells, we established a CRC cell model with low PPP2CA expression and performed cell phenotype experiments. Results showed that PPP2CA gene knockdown significantly promoted cell proliferation and migration in both HCT116 and SW480 cell lines, further confirming that PPP2CA knockdown enhances the malignant phenotype of CRC cells. Notably, our previous studies also revealed increased sensitivity to ferroptosis in cells after PPP2CA knockdown (7). In CRC cells, heightened sensitivity to ferroptosis is often associated with reduced malignant phenotype (9, 10). Thus, although knocking down PPP2CA (a gene with tumor-suppressive effects) increases cell susceptibility to ferroptosis, this increased susceptibility typically indicates decreased malignant phenotype of cells. This apparent paradoxical relationship has attracted our attention.

Studies have demonstrated that ferroptosis exerts a significant and complex regulatory effect on the development and progression of various tumors, including CRC. To further analyze the relationship between PPP2CA expression and ferroptosis in CRC cells, we first performed a comprehensive literature review to identify ferroptosis-related genes closely associated with CRC. Subsequently, we used bioinformatics databases to explore the associations between PPP2CA and these ferroptosis-related genes. Following this, we screened for ferroptosis-related genes significantly associated with PPP2CA and validated these genes in a PPP2CA-knockdown CRC cell model.​ Results showed that in the CRC cell line HCT116, the expression of the ferroptosis-related genes TFRC and ACSL4 was significantly upregulated. TFRC encodes the transferrin receptor (TfR), a protein critical for mediating cellular iron uptake and regulating ferroptosis in various tumor cells (16). By contrast, ACSL4 promotes lipid peroxidation via activating polyunsaturated fatty acids, thus contributing to the regulation of cellular ferroptosis (17). Thus, TFRC and ACSL4 expression is closely associated with cellular sensitivity to ferroptosis. We hypothesize that PPP2CA knockdown may regulate ferroptosis in CRC cells via upregulating TFRC and ACSL4.

To further analyze the potential regulatory relationships among PPP2CA, TFRC, and ACSL4, we performed an analysis using the STRING database. Results showed that PPP2CA could interact with TFRC via mTOR, and mTOR also interacts with the proteins encoded by ACSL4 and TFRC. mTOR is a key eukaryotic protein kinase involved in regulating multiple physiological processes, including cell growth, metabolism, and protein synthesis. By binding to different proteins, it can form two distinct complexes: mTOR complex 1 (mTORC1) and mTOR complex 2 (mTORC2) (18). Protein phosphatase 2A (PP2A) regulates mTOR, as it can dephosphorylate mitogen-activated protein kinase kinase kinase kinase 3 (MAP4K3)—a serine/threonine kinase family member and regulator of mTORC1—thereby inhibiting mTORC1 activity (19). Similarly, studies have demonstrated high mTOR activation in CRC (20). Given that the protein encoded by PPP2CA is the catalytic subunit of PP2A, and considering the close association between PPP2CA and mTOR identified via the STRING database, we hypothesize that PPP2CA regulates mTOR. In our subsequent experiments, we confirmed that PPP2CA knockdown promoted the upregulation of phosphorylated mTOR (p-mTOR) expression.

The mechanistic target of rapamycin (mTOR) exhibits close associations with ferroptosis-related genes, including transferrin receptor (TFRC) and acyl-CoA synthetase long-chain family member 4 (ACSL4). Studies have shown that mTOR activation in mouse embryonic fibroblasts enhances TfR expression, while the mTOR inhibitor rapamycin (RAPA) suppresses TfR expression (21). Furthermore, studies have demonstrated that co-exposure to cadmium and molybdenum activates the AMPK/mTOR axis in duck myocardial cells, leading to upregulation of ACSL4, PTGS2, and TFRC expression (22). In our study, we confirmed that PPP2CA knockdown increases phosphorylated mTOR (p-mTOR), which subsequently upregulates TfR and ACSL4 protein expression. This effect can be reversed by the mTOR inhibitor RAPA—further substantiating the hypothesis that PPP2CA regulates TFRC and ACSL4 via the mTOR signaling pathway.

In our previous studies, we observed that PPP2CA knockdown activated AMPK, which subsequently suppressed SCD1, thereby increasing cellular sensitivity to ferroptosis. Furthermore, in the present study, we found that AMPK activation induced by PPP2CA knockdown also triggered mTOR activation; this further enhanced cellular sensitivity to ferroptosis via upregulation of TFRC and ACSL4. Following PPP2CA knockdown, the malignant phenotype of CRC cells was exacerbated, while their sensitivity to ferroptosis increased. This phenomenon may be attributed to the ability of PPP2CA knockdown to regulate the biological behavior of tumor cells through multiple mechanisms, with the ultimate enhancement of the malignant phenotype resulting from the combined effects of these mechanisms (23). This study has several limitations. First, Ferroptosis is characterized by iron overload, the accumulation of reactive oxygen species, and a reduction in GPX4 levels (24, 25). However, this study did not explore these factors, which indicates an important avenue for further research. Second, cell experiments were only performed using two CRC cell lines (HCT116 and SW480), which may limit the generalizability of the findings to other molecular subtypes of CRC. Third, *in vivo* validation using nude mouse xenograft models or transgenic animal models has not been conducted, which represents a key direction for future research. Certainly, the mechanistic evidence linking PPP2CA and mTOR phosphorylation with the upregulation of TFRC and ACSL4 requires further experimental validation.

## 5 Conclusion

In summary, PPP2CA knockdown in CRC cells activates the AMPK/mTOR signaling pathway, leading to upregulation of the TFRC and ACSL4 genes, which in turn increases cellular sensitivity to ferroptosis. Furthermore, activated AMPK enhances ferroptosis sensitivity by downregulating SCD1. This indicates that ferroptosis in CRC cells following PPP2CA knockdown is a complex process regulated by multiple pathways. Similarly, after PPP2CA knockdown, cellular biological behaviors are subject to multifactorial regulation, and the increased ferroptosis sensitivity induced by PPP2CA knockdown is insufficient to reverse the malignant phenotype of the cells.

## Data Availability

The datasets presented in this study can be found in online repositories. The names of the repository/repositories and accession number(s) can be found in the article/supplementary material.
